# A model-based assessment of the cost–utility of strategies to identify Lynch syndrome in early-onset colorectal cancer patients

**DOI:** 10.1186/s12885-015-1254-5

**Published:** 2015-04-25

**Authors:** Tristan Snowsill, Nicola Huxley, Martin Hoyle, Tracey Jones-Hughes, Helen Coelho, Chris Cooper, Ian Frayling, Chris Hyde

**Affiliations:** 1 Institute of Health Research, University of Exeter Medical School, University of Exeter, Exeter, UK; 2 Institute of Cancer & Genetics, Cardiff University, Cardiff, UK

**Keywords:** Lynch syndrome, "Colorectal neoplasms, Hereditary Nonpolyposis" [MeSH], "Models, Economic" [MeSH], Cost–utility analysis

## Abstract

**Background:**

Lynch syndrome is an autosomal dominant cancer predisposition syndrome caused by mutations in the DNA mismatch repair genes *MLH1*, *MSH2*, *MSH6* and *PMS2*. Individuals with Lynch syndrome have an increased risk of colorectal cancer, endometrial cancer, ovarian and other cancers. Lynch syndrome remains underdiagnosed in the UK. Reflex testing for Lynch syndrome in early-onset colorectal cancer patients is proposed as a method to identify more families affected by Lynch syndrome and offer surveillance to reduce cancer risks, although cost-effectiveness is viewed as a barrier to implementation. The objective of this project was to estimate the cost–utility of strategies to identify Lynch syndrome in individuals with early-onset colorectal cancer in the NHS.

**Methods:**

A decision analytic model was developed which simulated diagnostic and long-term outcomes over a lifetime horizon for colorectal cancer patients with and without Lynch syndrome and for relatives of those patients. Nine diagnostic strategies were modelled which included microsatellite instability (MSI) testing, immunohistochemistry (IHC), *BRAF* mutation testing (methylation testing in a scenario analysis), diagnostic mutation testing and Amsterdam II criteria. Biennial colonoscopic surveillance was included for individuals diagnosed with Lynch syndrome and accepting surveillance. Prophylactic hysterectomy with bilateral salpingo-oophorectomy (H-BSO) was similarly included for women diagnosed with Lynch syndrome. Costs from NHS and Personal Social Services perspective and quality-adjusted life years (QALYs) were estimated and discounted at 3.5% per annum.

**Results:**

All strategies included for the identification of Lynch syndrome were cost-effective versus no testing. The strategy with the greatest net health benefit was MSI followed by *BRAF* followed by diagnostic genetic testing, costing £5,491 per QALY gained over no testing. The effect of prophylactic H-BSO on health-related quality of life (HRQoL) is uncertain and could outweigh the health benefits of testing, resulting in overall QALY loss.

**Conclusions:**

Reflex testing for Lynch syndrome in early-onset colorectal cancer patients is predicted to be a cost-effective use of limited financial resources in England and Wales. Research is recommended into the cost-effectiveness of reflex testing for Lynch syndrome in other associated cancers and into the impact of prophylactic H-BSO on HRQoL.

**Electronic supplementary material:**

The online version of this article (doi:10.1186/s12885-015-1254-5) contains supplementary material, which is available to authorized users.

## Background

Lynch syndrome (LS; previously known as hereditary non-polyposis colorectal cancer, HNPCC) is an autosomal-dominant cancer predisposition syndrome caused by mutations in the DNA mismatch repair (MMR) genes *MLH1*, *MSH2*, *MSH6* and *PMS2* [1]. LS predisposes to colorectal cancer (CRC) as well as extracolonic cancers including endometrial cancer and ovarian cancer (see Table 1).

**Table 1:** **Cumulative risk to age 70 of selected Lynch syndrome associated cancers**

Cancer	Risk to age 70	(95% CI)
Colorectal cancer (men)	38%	(25%–59%)
Colorectal cancer (women)	31%	(19%–50%)
Endometrial cancer (women)	33%	(16%–57%)
Ovarian cancer (women)	9%	(4%–31%)

Source: Bonadona et al. [45].

Notes: Estimates do not include *PMS2* mutation carriers.

Cancer prevention strategies can be employed for individuals with LS which benefit both individuals already affected by cancer and also those unaffected, yet LS remains underdiagnosed in the UK, in which there is no universal systematic testing for LS.

The National Institute for Health Research Health Technology Assessment Programme was asked to commission research into the cost-effectiveness of systematic testing for LS in individuals with newly diagnosed early-onset CRC and here we report the results of that research.

### Diagnosis

The diagnosis of LS rests on the results of microscopic and molecular tests. Microsatellite instability (MSI) in tumour tissue indicates a loss of MMR proficiency, while immunohistochemistry (IHC) of MMR proteins can indicate loss of their expression in a tumour; both indicate LS as a possible cause of the tumour. Sporadic tumours with MSI or lack of MMR protein expression also occur, so adjunctive tests such as for the *BRAF* V600E mutation and hypermethylation of the *MLH1* promotor can reduce false-positive results.

Although LS can be strongly suspected on the basis of personal and family history (such as the Amsterdam II criteria and revised Bethesda criteria) [1] allied with the results of tumour testing, ideally the finding of a pathogenic mutation in one of the DNA MMR genes is necessary for a firm diagnosis. The current standard for diagnostic testing for MMR mutations is DNA sequencing (to detect point mutations and small insertions/deletions) and multiplex ligation-dependent probe amplification (MLPA; to detect large structural DNA abnormalities). The finding of a pathogenic mutation is a prerequisite for predictive testing of relatives.

The interpretation of a mutation as pathogenic is complex and not always possible, although a significant recent advance has been made with a standardised classification scheme [2].

To avoid psychological harm, the genetic testing of individuals for constitutional mutations responsible for a cancer predisposition syndrome should only take place after informed consent and genetic counselling [3].

There are thousands of unique MMR DNA variants, although a proportion of these (around 11%) are not pathogenic or likely not pathogenic and a proportion (around 32%) have unknown significance (i.e., could be pathogenic but not confirmed) [2]. Screening for MMR mutations in unaffected individuals (i.e., in the general population) is generally thought to be prohibitively expensive and ill-advised due to the prevalence of variants of unknown significance and the lack of evidence regarding the psychological impact of such results. Screening is therefore reserved for individuals thought likely to have LS.

### Management

If LS is identified in an individual, surveillance can be offered to reduce the risk of CRC. UK guidelines state that “Total colonic surveillance (at least biennial) should commence at age 25 years. […] Surveillance should continue to age 70–75 years or until comorbidity makes it clinically inappropriate”. [4] High quality data from randomised trials is not available regarding the effectiveness of colonoscopic surveillance, but the best available published evidence suggests a 62% reduction in the risk of CRC for individuals with LS undergoing 3-yearly colonoscopy in Finland [5,6]. Despite the evidence showing that colorectal surveillance is effective in LS, recent work shows that there is poor compliance in the UK with international guidelines, with inadequate assessment and wide variability in the management of LS [7].

Evidence is lacking to support prophylactic surgery to prevent CRC or the practice of aggressive surgery (removing significantly more of the colorectum than necessary for treatment alone) for CRC, although the latter is recommended in the BSG/ACPGBI guidelines [4]. Likewise evidence is lacking to support surveillance for gynaecological cancers yet this too is recommended in guidelines [6]. There is evidence to support prophylactic surgery (H-BSO) to prevent gynaecological cancers [8], although impact on health-related quality of life (HRQoL) has not been assessed; guidelines have not recommended prophylactic surgery but have suggested it be presented as an option [6]. Recommendations are also made regarding surveillance for other cancers associated with LS, but without supporting evidence [6].

### Objective

To estimate the cost–utility of strategies to identify LS in early-onset CRC (aged under 50 years) in the NHS in England and Wales.

## Methods

We developed a decision analytic model in consultation with clinical experts (co-author Dr Ian Frayling; acknowledged contributors Mr Ian Daniels, Dr Carole Brewer and Mr John Renninson, all of Royal Devon & Exeter NHS Trust) and parameterised using the best available data relevant to the NHS.

### Population

Individuals in England and Wales newly diagnosed with CRC aged under 50 years (denoted probands) and their relatives, who would be offered predictive genetic testing if a LS mutation was found in a proband.

### Interventions

Nine diagnostic strategies for LS were chosen on the basis of the tests available, strategies in previous cost-effectiveness models and expert advice. Due to the lack of clearly defined current practice, two strategies were included in which genetic testing is not offered; in the first of these no attempt was made to identify LS in the probands, and in the second the Amsterdam II criteria were used. The final set of strategies was:

1. Strategies without genetic testing
  1(1). No testing at all (all diagnosed LS negative)
  2(2). Amsterdam II criteria for diagnosis
2. IHC four-panel test for *MLH1*, *MSH2*, *MSH6* and *PMS2*, followed by mutation testing if IHC result abnormal
3. IHC four-panel test, followed by *BRAF* V600E mutation testing if *MLH1* abnormal and mutation testing if MMR protein other than *MLH1* abnormal or *BRAF* V600E mutation not found
4. MSI testing, followed by mutation testing if MSI found
5. MSI testing, followed by *BRAF* V600E mutation testing if MSI found, followed by mutation testing if *BRAF* V600E mutation not found
6. As Strategy 5 but IHC performed in parallel with mutation testing to aid interpretation (i.e., not used diagnostically)
7. IHC four-panel test followed by mutation testing if IHC result abnormal. If IHC result normal, follow Strategy 5
8. Direct mutation testing.

Mutation testing for LS includes both sequencing and MLPA. Probands would be classified as LS positive if a mutation was found or LS assumed if no mutation was found but LS was suspected on the basis of family history. In addition probands could decline genetic counselling or diagnostic genetic testing and in this case would be classified as LS assumed or LS negative on the basis of family history.

When LS mutations were found in probands, testing was offered to their first-degree relatives (FDRs). Where the family mutation was also found in those FDRs, cascade testing was used to reach more distant relatives. When probands were assumed to have LS, only their FDRs were assumed to have Lynch syndrome.

Individuals classified as LS positive or LS assumed would be offered biennial colonoscopic surveillance commencing at age 25 and ending at age 75.

### Outcomes

The primary outcomes were the expected total costs and quality-adjusted life years (QALYs) for each diagnostic strategy, the incremental cost-effectiveness ratios (ICERs) of the strategies and the incremental net health benefit (INHB) of the strategies at a willingness-to-pay of £20,000 per QALY.

Secondary outcomes were the diagnostic test accuracies of the strategies, the expected number of colonoscopies and cancers in each strategy, and the life expectancy in each strategy.

### Study design

We developed a decision analytic model with two components.

The first component (the diagnostic submodel) consisted of a decision tree and was used to estimate the number of probands and relatives who would receive each possible diagnosis and to estimate how many individuals diagnosed with Lynch syndrome would accept colonoscopic surveillance for each of the strategies listed in *Interventions (above)*. It also calculated the cost of diagnosis in each strategy.

The second component (the management submodel) consisted of an individual patient simulation and was used to estimate the lifetime costs that would be incurred through colonoscopies, CRC treatment, hysterectomies (note these also include bilateral salpingo-oophorectomy) and endometrial cancer treatment and the life years and QALYs that would be accrued for individuals with each diagnosis.

The results of the two submodels were combined to give a full incremental analysis of costs and QALYs [9].

The management submodel included a number of possible events: colonoscopy, colonoscopy complication, colonoscopy mortality, CRC incidence, metachronous CRC incidence, CRC mortality, prophylactic hysterectomy, prophylactic hysterectomy mortality, endometrial cancer incidence, endometrial cancer mortality and general mortality. These events determined the costs incurred and life years and QALYs accrued.

In line with the NICE reference case [10], the perspective of NHS and Personal Social Services was adopted and costs and benefits were discounted by 3.5% per annum. Costs were converted to pounds sterling (£) using purchasing power parities [11] (where appropriate) and adjusted to 2013/14 prices using the Hospital and Community Health Services (HCHS) index [12]. Individuals were simulated up to age 100 or until death.

Parameters relating to the natural histories of diseases, the effectiveness of interventions and the impact on HRQoL of diseases and interventions were sourced, where possible, from national statistics and published literature. Where such values were not available, estimates were sought from clinical experts, with priority given to clinical data.

If data permitted, diagnostic test accuracy parameters were estimated according to previous tests, e.g., separate estimates of the test accuracy of *BRAF* V600E mutation testing were used depending on whether IHC or MSI was the preceding test. In some cases such estimates were not available and it was necessary to assume the independence of diagnostic tests.

Colonoscopy was estimated to reduce the incidence of index CRC (i.e., the first incident CRC in an individual) using a hazard ratio of 0.387 estimated from a Finnish cohort study [5]. Surveillance colonoscopy was estimated to reduce the incidence of metachronous CRC (i.e., a subsequent incident CRC) using a hazard ratio of 0.533 estimated from an Italian cohort study [13]. Individuals were assumed to develop a maximum of two CRCs over a lifetime. Colonoscopies were received every three years in the Finnish cohort study [5] but occur every two years in our decision model. The effectiveness of biennial colonoscopy may therefore be underestimated and we adjusted the cost of colonoscopies down by a third to remove this bias (but keep true representations of the number of colonoscopies and the associated risks). Colonoscopies were received every two years in the Italian cohort study [13] but the same cost (reduced by a third) was used for colonoscopies intended to prevent metachronous CRC, which would bias cost-effectiveness in favour of interventions. Sensitivity analyses investigated the effect of colonoscopies being more costly and of surveillance being less effective.

General population norms for HRQoL were included based on Ara and Brazier [14]. No disutility was assumed for individuals with CRC unless they had metastatic cancer [15] (Dukes’ stage D), in which case a disutility of 0.13 was applied [16]. Colonoscopy was assumed not to affect HRQoL. Different types of colorectal surgery were modelled but no HRQoL difference was included in the base case [17]. No disutility was assumed for endometrial cancer as most patients would be diagnosed with early stage cancer [8] and a study of 264 women found HRQoL was similar for early stage endometrial cancer patients as for those in the general population [18]. No disutility was assumed for prophylactic H-BSO as it is not offered until childbearing would be expected to be completed. Disutilities were applied to account for the psychological impact of genetic testing on HRQoL for four months [19].

Additional file 1 gives further details about our modelling approach for interested readers and to allow completion of the CHEERS checklist [20] in Additional file 2.

Additional file 3: Tables S1 and S2 detail and give sources for the model parameters of the diagnostic and management components respectively.

## Results

### Base case results

All strategies except Strategies 1(2) (family history only) and 8 (direct mutation testing) had specificity over 99.5% in relation to probands. All strategies except Strategy 1(2) had sensitivity of 60% or greater. Strategy 3 had the highest positive predictive value (98.7%) and Strategy 7 had the highest negative predictive value (97.8%). The use of *BRAF* V600E testing in strategies improved specificity without compromising sensitivity.

**Figure 1:**
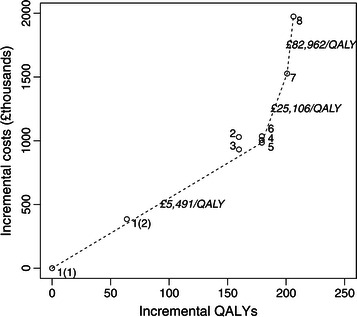
Cost–utility plane (base case results, representing an annual cohort from England).

Table 2 gives the cost–utility results in the base case and these are shown on the cost–utility plane in Figure 1. Secondary outcomes across strategies are given in Table 3.

**Table 2:** **Base case results representing an annual cohort from England (primary outcomes)**

Strategy	1(2)	2	3	4	5	6	7	8
*Incremental costs vs Strategy 1(1) [£ Thousands]*								
Diagnosis	48.9	662.7	578.5	599.6	586.0	636.9	1061.6	1336.6
CRC prevention	396.7	735.9	726.9	822.1	817.1	817.1	928.8	1065.7
CRC treatment	−249.3	−646.9	−646.2	−725.5	−725.2	−725.2	−814.0	−848.8
EC prevention	210.4	338.1	333.2	377.3	374.5	374.5	427.0	499.6
EC treatment	−21.7	−60.6	−60.6	−68.0	−68.0	−68.0	−76.2	−78.7
**Total**	**384.9**	**1029.2**	**931.8**	**1005.4**	**984.5**	**1035.3**	**1527.1**	**1974.5**
*Incremental QALYs vs Strategy 1(1)*								
Short-term	0	−4.3	−4.1	−4.8	−4.6	−4.6	−5.5	−8.5
Long-term	63.9	164.0	163.9	184.0	183.9	183.9	206.4	214.8
**Total**	**63.9**	**159.7**	**159.8**	**179.2**	**179.3**	**179.3**	**200.9**	**206.3**
*Cost–utility*								
ICER vs Strategy 1(1) [cost per QALY gained]	£6021	£6444	£5831	£5610	£5491	£5774	£7601	£9571
ICER [cost per QALY gained]	ED	D	ED	D	**£5491**	D	**£25106**	**£82962**
INHB at WTP £20000/QALY vs 1(1) [QALYs]	44.7	108.3	113.2	129.0	130.1	127.5	124.5	107.6

Key: D, dominated; EC, endometrial cancer; ED, extended dominated; WTP, willingness-to-pay.

**Table 3:** **Base case results representing an annual cohort from England (secondary outcomes)**

Strategy	1(2)	2	3	4	5	6	7	8
Number of colonoscopies vs Strategy 1(1) (=4162)	+1618	+3044	+3008	+3401	+3381	+3381	+3842	+4400
Life expectancy of index patient vs Strategy 1(1) (=13.82 years)	+0.06	+0.10	+0.10	+0.12	+0.12	+0.12	+0.13	+0.14
Life expectancy of index patient with LS vs Strategy 1(1) (=12.93 years)	+0.72	+1.24	+1.24	+1.39	+1.39	+1.39	+1.56	+1.61
Life expectancy of relative vs Strategy 1(1) (=37.38 years)	+0.01	+0.05	+0.05	+0.05	+0.05	+0.05	+0.06	+0.06
Life expectancy of relative with LS vs Strategy 1(1) (=33.97 years)	+0.31	+1.24	+1.24	+1.39	+1.39	+1.39	+1.56	+1.61
Expected number of CRCs vs Strategy 1(1) (=664.9)	−8.36	−24.59	−24.56	−27.59	−27.57	−27.57	−30.95	−32.30
Expected number of ECs vs Strategy 1(1) (=53.8)	−4.99	−14.29	−14.29	−16.03	−16.03	−16.03	−17.97	−18.55

*Abbreviations*: *EC* endometrial cancer.

Total discounted costs (across the population for an annual cohort) ranged from £36.22 m for Strategy 1(1) to £38.20 m for Strategy 8. The use of *BRAF* V600E testing reduced total discounted costs as savings were made in the number of diagnostic genetic tests.

Total discounted QALYs (across the population for an annual cohort) ranged from 151,793 for Strategy 1(1) to 152,000 for Strategy 8. The use of BRAF V600E testing slightly improved total discounted QALYs (<0.1 QALYs across population).

Strategies 2, 4 and 6 were dominated by (i.e., were more costly and less effective than) another strategy. Strategies 1(2) and 3 were extended dominated, i.e., were more costly and less effective than some combination of other strategies. Strategies 1(1), 5, 7 and 8 were therefore on the cost-effectiveness frontier (neither dominated nor extended dominated), as shown in Figure 1. The ICER of Strategy 8 versus Strategy 7 was £82,962/QALY, substantially greater than the UK cost-effectiveness threshold of £20,000 per QALY, suggesting that direct reflex mutation testing is not cost-effective in early-onset CRC patients. At a willingness-to-pay of £20,000 per QALY, Strategy 5 resulted in the greatest net health benefit (in which costs are converted to QALY losses and offset against QALY gains) of 130.1 QALYs versus Strategy 1(1). Compared to Strategy 1(1), all strategies had an ICER under £10,000/QALY and are therefore considered cost-effective versus no testing.

### Scenario analyses

We conducted scenario analyses investigating the impact of altering the inclusion age range for reflex testing for LS and of replacing *BRAF* testing with *MLH1* methylation testing. We also conducted univariate sensitivity analyses on a number of parameters.

### Expanding the inclusion age range

We explored the impact of increasing the inclusion age from 50 years to 60 years and to 70 years. A number of parameters were altered for consistency, most notable of these being: the number of probands offered reflex testing was increased from 1,699 in the base case to 5,018 and 13,900 as CRC patients aged under 60 and 70 years respectively are included; the prevalence of LS in the probands was reduced from 8.4% in the base case to 5.7% and 3.8%.

In both scenarios Strategy 5 remained the most cost-effective strategy at a willingness-to-pay of £20,000 per QALY (Table 4). In both scenarios there was little difference in QALY gain between Strategy 7 and Strategy 8 but there were significant cost increases associated with Strategy 8 which suggest universal reflex mutation testing would definitely not be cost-effective in older CRC patients. Strategy 5 remained cost-effective even when the cost of colonoscopies was doubled which suggests these results are fairly robust.

**Table 4:** **Cost–utility when age limit is raised to 60 and 70 years (representing an annual cohort from England)**

Scenario	Base case (CRC under 50 years)	CRC under 60 years	CRC under 70 years
*Incremental costs of Strategy 5 vs Strategy 1(1) [£ Thousands]*			
Diagnosis	586.0	1590.5	4132.2
CRC prevention	817.1	1630.3	2990.5
CRC treatment	−725.2	−1450.7	−2604.6
EC prevention	374.5	772.5	1430.4
EC treatment	−68.0	−139.2	−247.9
**Total**	**984.5**	**2403.4**	**5700.5**
*Incremental QALYs Strategy 5 vs Strategy 1(1)*			
Short-term	−4.6	−9.5	−18.1
Long-term	183.9	322.4	574.4
**Total**	**179.3**	**312.9**	**556.3**
*Cost–utility of Strategy 5 vs Strategy 1(1)*			
ICER [cost per QALY gained]	£5491	£7681	£10247
INHB at WTP £20,000/QALY [QALYs]	130.1	192.8	271.3

*Abbreviations*: *EC* endometrial cancer, *WTP* willingness-to-pay.

The INHB obtained when an age limit of 70 years was used exceeded the INHBs for age limits of 50 and 60 years due to the increased population size. On average less benefit would be accrued for each individual (and more CRC patients without LS would be subjected to some amount of testing), but in aggregate results suggest an age limit of 70 years is cost-effective.

### Replacing *BRAF* mutation testing with *MLH1* hypermethylation testing

*MLH1* promotor hypermethylation causes microsatellite instability and can masquerade as LS [21]. The detection of *MLH1* promotor hypermethylation can be used to rule out LS unless other risk factors are present (e.g., early-onset CRC, family history).

We conducted a scenario analysis in which *BRAF* testing in strategies was replaced by methylation testing. We found that in this scenario ICERs versus Strategy 1(1) did not change significantly from in the base case, but there were small changes to costs and QALYs which changed the strategies on the cost-effectiveness frontier. In this scenario Strategy 4 now gives more QALYs than Strategy 5 (and remains more expensive) and is therefore no longer dominated. Strategy 4 in fact now gives the greatest INHB at a willingness-to-pay of £20,000 per QALY (129.0 QALYs), although this is still lower than the INHB of Strategy 5 in the base case, which suggests that methylation testing may not be as cost-effective as *BRAF* testing, although the difference (if there is one) is likely to be small.

### Univariate sensitivity analyses

We conducted univariate sensitivity analyses on the majority of parameters (results are presented as tornado diagrams in Additional file 1). Strategy 5 remained cost-effective versus Strategy 1(1) in all sensitivity analyses except when prophylactic H-BSO was assumed to reduce utility by 0.1, in which case Strategy 1(1) (no testing) dominated all strategies, i.e., it was the least costly and gave the most QALYs. Strategy 8 (direct mutation testing) was found to be cost-effective when the costs of mutation tests for probands were halved – this gives an indication that as costs of mutation testing decrease (including through next generation sequencing), tumour-based tests IHC, MSI and *BRAF* V600E may no longer be necessary for cost-effective diagnosis. Notably, another sensitivity analysis suggested that reflex testing remains cost-effective even when no relatives are identified for testing, with an ICER of £6,725/QALY (higher than the £5,491/QALY base case with five relatives identified but still below the £20,000/QALY cost-effectiveness threshold). The relative robustness of our results to this parameter is due to the inclusion of significant risk-reducing measures for metachronous cancer (colorectal and endometrial) in the proband and because we find that testing in relatives leads to increased costs as well as improved outcomes.

### Impact on colonoscopy services

**Figure 2:**
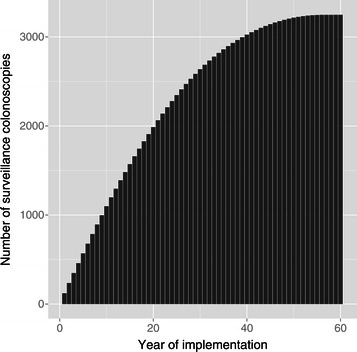
Projected number of surveillance colonoscopies if Strategy 5 were to be introduced in England.

If Strategy 5 were introduced in England, we project that the number of surveillance colonoscopies would increase until a steady state of approximately 3,250 surveillance colonoscopies would be conducted per year, with an initial growth rate of approximately 120 surveillance colonoscopies per year (Figure 2). The steady state corresponds to approximately two surveillance colonoscopies for each proband tested per year with an initial growth rate of approximately one surveillance colonoscopy for each 14 probands tested. These projections are based on the assumptions of no demographic or epidemiological changes.

For example, a district general hospital serving a population of 400,000 would expect to reach a steady state of approximately 25 surveillance colonoscopies per year, with an initial growth rate of approximately one colonoscopy per year. This would probably be a small impact on colonoscopy services compared to interventions such as the NHS Bowel Cancer Screening Programme, for which approximately 1,000 colonoscopies are conducted each week in the UK [22], corresponding to approximately 300 colonoscopies per year for the hypothetical district general hospital.

A number needed to treat calculation suggests that approximately 90 additional colonoscopies would be needed to prevent one CRC if Strategy 5 were to be introduced. Colonoscopies also identify CRCs in early stages, thereby improving survival. The combination of these and other factors suggest 4–5 colonoscopies would be needed to save one life year, and 6 colonoscopies would be needed to save one QALY.

## Discussion

### Relation to previous findings

There are no comparable studies in the NHS but others have evaluated the cost-effectiveness of strategies to identify LS elsewhere. These have generally adopted a similar approach to ours – the identification of LS in early-onset CRC patients and in their relatives. Our results are broadly consistent with those of others that age-targeted testing for LS with preliminary tests before diagnostic mutation testing is cost-effective versus no testing [23-28]. There is some disagreement whether direct diagnostic mutation testing would be cost-effective versus no testing, but all studies agree that it would not be cost-effective versus strategies with preliminary tests. Our results also suggested that reflex testing would be cost-effective even if relatives cannot be identified for testing, while some previous analyses have identified this as a very sensitive parameter for cost-effectiveness [25-28]. Some of these failed to include any potential direct benefits to probands in terms of prevention of metachronous cancer [25,26]. Dinh, Rosner et al. considered the approach of general population screening using a risk prediction tool (PREMM_126_) [29] in primary care with a comparator arm of “current practice” and found that screening individuals at various ages with a predicted risk of carrying LS of 5% or greater was cost-effective [30]. It is not clear whether such a strategy would be cost-effective versus systematic reflex testing as proposed in this analysis, since current practice was assumed to include low awareness of Lynch syndrome and limited availability of IHC and MSI. While the approach of Dinh, Rosner et al. could result in faster identification of families with Lynch syndrome than reflex testing of CRC patients, it would also entail a significant and possibly disruptive burden on primary care when initiated, which also may not have been costed in their analysis.

### Strengths and limitations

We did not include costs of surveillance for gynaecological cancer, although this has been recommended in clinical guidance [31], because we did not find evidence of the effectiveness of such surveillance, and clinical opinion we have sought suggests it is not effective at identifying ovarian cancer and not always used in practice. Given that this surveillance can be costly (estimated at over $350 per year [32]), it would seem prudent to evaluate the effectiveness and cost-effectiveness of gynaecological surveillance before recommending it to women with LS, particularly as it may displace prophylactic surgery which has been shown to be effective in preventing gynaecological cancers [8].

We have not modelled ovarian cancer or other cancers associated with LS. Ovarian cancer affects fewer individuals with LS than CRC or endometrial cancer [33] but is associated with poor survival [34], so it is likely that failure to model ovarian cancer underestimates the benefits of prophylactic bilateral salpingo-oophorectomy (which is already modelled as a cost for endometrial cancer prevention) and in this respect our analysis underestimates the cost-effectiveness of testing for LS. The risks of other cancers associated with LS are highly uncertain and it is not clear whether risk-reducing measures such as surveillance are effective or could be cost-effective for these cancers.

We have not included chemoprevention in our analysis, although recent developments suggest that chemoprevention may have a role in the management of individuals with LS [31], with the CAPP2 study strongly suggesting a reduction in the risk of associated cancers in individuals with LS due to long-term aspirin [35] and the Petals trial designed to investigate the effectiveness of LNG-IUS in preventing endometrial cancer in individuals with LS [36]. These are low cost interventions which would very likely be cost-effective if clinical benefit is confirmed and quantified.

When considering the generalizability of our analysis it is important to consider infrastructure requirements to ensure that testing is completed and the results used appropriately. While much of this infrastructure already exists in the UK (particularly for testing) there may be local variation in follow-up and surveillance after diagnosis.

### Areas of uncertainty

Uncertainty exists regarding the impact of prophylactic H-BSO on HRQoL; in a sensitivity analysis this was found to have a drastic effect on cost–utility (since it is offered to so many individuals in the population), resulting in Strategy 1(1) (no testing) being less costly and more effective than all others. If it is thought that a disutility of 0.1 is plausible, studies should be conducted to estimate the true impact on HRQoL as a priority. We note that the best source for utility values following hysterectomy identified in a recent economic study [37] involving hysterectomy (in this case for menorrhagia) was a Finnish study using EQ-5D to compare the levonorgestrel-releasing intrauterine system (LNG-IUS) with hysterectomy over five years [38]. Participants randomised to hysterectomy in this study had an average “EQ-5D index” of 0.88 five years after randomisation. While the EQ-5D index is not a preference-based utility value (it is instead a regression model mapping EQ-5D states to EQ-5D VAS measurements), it is scaled 0–1 and the Finnish female population appears to measure at 0.91 for ages 35–44 and 0.89 for ages 45–54 [39], which suggests that the long-term disutility of hysterectomy is likely to be small. It may be possible that the addition of bilateral salpingo-oophorectomy results in reduced HRQoL which is not measured by Hurskainen et al. since only 7/109 participants received bilateral salpingo-oophorectomy and results for these participants are not presented separately [38].

### Other considerations

Next generation sequencing may lead to significant cost reductions in mutation testing for LS, meaning that in the future direct mutation testing may be cost-effective. In the past there have been concerns that direct mutation testing could lead to significant psychological harms but recent improvements in the classification of variants of uncertain significance in LS [2] and the encouraging interim results from the Mainstreaming Cancer Genetics programme [40] could result in a shift towards direct mutation testing for hereditary cancer syndromes such as LS.

A very recent development in tumour testing for LS is the use of IHC to detect *BRAF* V600E mutations, the performance of which has been demonstrated to varying degrees [41,42]. If sufficient diagnostic performance can be obtained from this test it may be possible to perform sensitive and specific tumour-based testing for LS purely using IHC and avoiding the cost of molecular genetic tests.

Microsatellite instability has been shown to be predictive of the efficacy of fluorouracil-based adjuvant chemotherapy [43], which has led to suggestions that MMR proficiency should be evaluated in all CRC patients who might receive adjuvant chemotherapy (Stage II and above). If testing for MMR proficiency becomes widespread then the incremental cost of testing for LS will decrease (since some tumour-based testing will already have been conducted for many patients).

## Conclusions

The results presented suggest that reflex testing for LS would be a cost-effective use of limited NHS resources and in the base case of testing in newly-diagnosed CRC patients aged under 50 years would not create an excessive additional burden for colonoscopy services. As cost-effectiveness may be a perceived barrier to the introduction of reflex testing, these results may result in national policy change.

Maximum net health benefit was estimated to be obtained when all newly-diagnosed CRC patients aged under 70 years are tested. Reflex testing remained cost-effective even when the cost of colonoscopies (one of the most sensitive parameters) was doubled, which suggests there is some robustness in this conclusion. Decision makers will likely want to consider all sources of uncertainty and also consider budget impact and the impact on colonoscopy services of any policy changes.

We recommend further research to establish whether it is cost-effective to perform reflex testing in other LS-associated cancers (such as endometrial and ovarian cancer). We also recommend a controlled study of HRQoL in women following prophylactic H-BSO using the EQ-5D tool to ensure that this does not lead to an overall loss of QALYs. We further recommend that the effectiveness of gynaecological surveillance for endometrial and ovarian cancer in women with LS is evaluated.

## Research ethics

No human subjects, human material, or human data were involved in this research, which is based on literature review and software modelling.

## Additional files

Additional file 1: **Support for CHEERS checklist.** Provides additional information to support the CHEERS checklist (Additional file 2), including further details of the decision analytic model and tornado diagrams for univariate sensitivity analyse.
Additional file 2: **CHEERS checklist.** Gives references to where in this document or in the other additional files the CHEERS checklist items are satisfied.
Additional file 3: **Supplementary Tables.** Provides supplementary tables of model parameter values and sources for the decision analytic model.

## Abbreviations

ACPGBI: Association of Coloproctology of Great Britain and Ireland
BSG: British Society of Gastroenterology
CAPP2: Colorectal adenoma/carcinoma prevention programme
CRC: Colorectal cancer
EQ-5D: EuroQol five dimensions
FDR: First-degree relative
H-BSO: Hysterectomy and bilateral salpingo-oophorectomy
HCHS: Hospital and Community Health Services
HRQoL: Health-related quality of life
ICER: Incremental cost-effectiveness ratio
IHC: Immunohistochemistry
INHB: Incremental net health benefit
LNG-IUS: Levonorgestrel-releasing intrauterine system
LS: Lynch syndrome
MLPA: Multiplex ligation-dependent probe amplification
MMR: DNA mismatch repair
MSI: Microsatellite instability
QALY: Quality-adjusted life year
VAS: Visual analog scale
