# Supplementary material for: A model-based assessment of the cost–utility of strategies to identify Lynch syndrome in early-onset colorectal cancer patients
Source: BMC Cancer. 2015 Apr 25;15:313. doi: 10.1186/s12885-015-1254-5 (PMC4428233; doi:10.1186/s12885-015-1254-5)
Supplement: Additional file 1: — Support for CHEERS checklist. Provides additional information to support the CHEERS checklist (Additional file 2), including further details of the decision analytic model and tornado diagrams for univariate sensitivity analyse. [file 12885_2015_1254_MOESM1_ESM.docx]

# Additional file 1 – Support for CHEERS checklist

Here we provide additional information to support the CHEERS checklist [[1](#_ENREF_1), [2](#_ENREF_2)] for reporting economic evaluations which could not be included in the main report due to space restriction.

## Broader context, original question and relevance for health policy or practice decisions

This project was commissioned by the National Institute for Health Research (NIHR) Health Technology Assessment (HTA) programme. The HTA programme identifies and prioritises topics for research to meet the needs of the UK National Health Service (NHS).

The original question the project was intended to answer was, “What is the diagnostic utility and cost-effectiveness of genetic testing for Hereditary Non Polyposis Colorectal Cancer (HNPCC) in all newly diagnosed patients with colorectal cancer under 50 years of age, and of strategies to test their close relations?”

The project is relevant to clinical practice because at present there is no UK national policy on identifying Lynch syndrome. While this project was not specifically designed to inform a national policy decision, it is expected to affect clinical practice. In addition to relevance to UK practice, we have also been in contact with groups in Spain, Germany and Australia who are keen to use the findings of this project to support changes in policy or practice or additional research.

## Setting and location

The setting for most interventions in the economic evaluation is secondary/tertiary care. Surgery for cancer and prophylaxis are conducted in hospitals, as are surveillance colonoscopies. Clinical genetics services (including genetic counselling) are a part of tertiary care. Few interventions are in primary care, but these could include general practitioner appointments leading to cancer diagnosis.

The location for the economic analysis is the United Kingdom. Many parameters were sourced in particular from England (84% of UK population) or England and Wales (89% of UK population).

## Perspective

The costs of interventions were derived from the perspective of the NHS and Personal Social Services. This includes relevant costs to government but not costs to patients. Productivity costs are not included. This is the standard perspective for health technology assessment in the UK and used as the reference case by the National Institute for Health and Care Excellence (NICE) [[3](#_ENREF_3)].

## Measurement of effectiveness

No single study has addressed the research question or provided means of fully parameterising a model to address the research question. Several key works have attempted to synthesise evidence pertaining to many aspects of the research question. The EGAPP supplementary evidence review [[4](#_ENREF_4)] provided estimates for a number of parameters, particularly the diagnostic test accuracy of tumour-based tests. This is an update of an earlier review by Bonis et al. [[5](#_ENREF_5)]. We additionally searched previous economic models for parameters, in particular those by Dinh et al. [[6](#_ENREF_6)], Ladabaum et al. [[7](#_ENREF_7)], and Mvundura et al. [[8](#_ENREF_8)].

Where these reviews and models did not provide parameters deemed satisfactory, opportunistic searching of the published literature was conducted. In some cases, clinical expert opinion was the only identified evidence, particularly for parameters relating to compliance to or acceptance of interventions. Parameter sources are given in Additional file 3: Tables S1 and S2.

## Measurement and valuation of preference-based outcomes

Baseline utility (for individuals not affected by any included diseases or interventions) is based on Model 1 reported by Ara and Brazier [[9](#_ENREF_9)]. This model is based on data from the Health Survey for England 2003 and the Health Survey for England 2006, incorporating EQ-5D questionnaire responses from 26,679 people. The EQ-5D measurements were valued using time-trade-off valuations from the UK general public [[10](#_ENREF_10)]. This baseline utility allows for incorporation of general deterioration of health-related quality of life with age.

Short term disutility due to the psychological impact of genetic testing for Lynch syndrome was estimated on the basis of a single study by Kuppermann et al. [[11](#_ENREF_11)]. In this study, 70 individuals gave valuations of health state vignettes using the time-trade-off method. The individuals were from the USA and drawn from two sources. The first source was a general medical clinic (n=49) and the second was the Gastrointestinal Cancer Prevention Program (n=21); both of these sources were at a university hospital in the USA. Individuals from the Gastrointestinal Cancer Prevention Program were knowledgeable about and at high risk for Lynch syndrome. Overall the study population was more highly educated than the US general population. Rather than health states being measured by individuals experiencing genetic testing for Lynch syndrome (e.g., using EQ-5D), instead health state vignettes were created with no reporting of their development. The use of health state vignettes is not recommended by NICE, which also recommends that health states should be valued by the UK general public. The study population was not representative of the UK general public but this was believed to be the best available evidence.

Disutility due to colorectal cancer (CRC) was estimated as 0 for non-metastatic CRC and 0.13 for metastatic CRC. Cooper et al. recently conducted a systematic review of health-related quality of life in patients with CRC as part of an economic analysis for chemoprevention of CRC [[12](#_ENREF_12)]. They identified a number of studies and concluded that a study by Ness et al. [[13](#_ENREF_13)] gave the best estimates for the utility of CRC states according to stage at diagnosis. We disagree with their conclusion since the health states were not measured in people actually experiencing them, nor were they measured using a generic health-related quality of life questionnaire. Furthermore they were not valued by the general public and the study population was fairly small.

We preferred to use estimates from the study by Ramsey et al. [[14](#_ENREF_14)]. In this study health states were measured in people experiencing them using the Health Utilities Index (HUI) Mark III. These health states were then valued using weights derived from valuation by the general public [[15](#_ENREF_15), [16](#_ENREF_16)]. Ramsey et al. did not find a statistically significant effect of CRC stage at diagnosis on utility. Unfortunately Ramsey et al. did not include a control group to judge the effect of CRC on utility as a whole, but we observed that the mean utility reported in the study was 0.85. Mapping this mean utility from HUI3 to EQ-5D gives a utility of 0.87 utilising the formula from Richardson et al. [[17](#_ENREF_17)]. This is a higher utility than that measured in the general population of England and Wales for individuals aged 71 (0.79 for men and 0.77 for women) [[9](#_ENREF_9)] and for this reason we did not apply a disutility for non-metastatic CRC.

Although the study by Ramsey et al. did not suggest a disutility for metastatic CRC, it was felt that this should be investigated further as metastatic cancer often results in disutility. Mittmann et al. report utility of 0.68 for patients in the best supportive care arm of an RCT of cetuximab [[18](#_ENREF_18)]. The mean age in this study was 64 and 64% of the patients were male. The general population at age 64 has estimated utility of 0.82 for males and 0.80 for females [[9](#_ENREF_9)]. A disutility of 0.13 (equal to 0.81 minus 0.68) was chosen for metastatic CRC on the basis of this analysis.

No additional disutility was applied for surgery for CRC. This is largely justified as any disutility due to surgery would already be measured in the disutility due to CRC, since the vast majority of patients would have been treated surgically. This does mean that more aggressive surgery for CRC is assumed to always be clinically superior to standard surgery, since the risk of metachronous CRC is reduced with no impact on quality of life. More research is required to measure the impact of different surgery for CRC using a generic health questionnaire (preferably EQ-5D).

No disutility was applied for endometrial cancer or for hysterectomy and bilateral salpingo-oophorectomy (H-BSO). This is because we found no high-quality data on relevant health-related quality of life and the two studies we did identify only investigated Stage I-II endometrial cancer and found health-related quality of life was similar to that of age-matched general populations. More research is required to measure the impact of endometrial cancer and H-BSO on health-related quality of life.

No disutility was applied for surveillance colonoscopies as it was assumed these would have a negligible effect on QALYs since the procedure is short. If the utility of colonoscopy was worse than death (i.e., less than 0) there could conceivably be an impact on total QALYs from surveillance colonoscopies but as these are generally described as uncomfortable rather than painful and distressing [[19](#_ENREF_19)] we feel the assumption is reasonable.

No disutility was applied for adverse events resulting from colonoscopy, e.g., bleeding and perforation, because these events were transient and rare and therefore unlikely to impact total QALYs.

## Resource use and unit costs

Most resource use in the model was trivial, e.g., resource use for surgery = 1, resource use for biennial colonoscopy = 1 per 2 years. Resource use for diagnostic tests was 0 if a test was not used in a strategy and 1 if the test was used in a strategy. Resource use for genetic counselling was assumed to be 75 minutes with a Band 7 counsellor and 30 minutes with a Band 5 clinic nurse (to take family history). Resource use for treatment of adverse events was driven by the frequency of adverse events. Resource use for treatment of CRC was assumed to be the same as that used in Trueman et al. [[20](#_ENREF_20)] and hence only unit costs were updated for this.

Resource uses for treatments of endometrial cancer were estimated as follows. Resource use of one for endometrial cancer surgery was assumed. Radiotherapy was measured in total courses, such that patients receiving radiotherapy incurred a resource use of one, patients not receiving radiotherapy incurred a resource use of zero. A third of individuals with Stage I endometrial cancer were assumed to have radiotherapy, as were all individuals with Stage II–III endometrial cancer and half of individuals with Stage IV endometrial cancer [[21](#_ENREF_21)]. Chemotherapy was assumed to be combination chemotherapy comprising doxorubicin 45 mg/m² IV plus cisplatin 50 mg/m² IV plus paclitaxel 160 mg/m² per cycle (over two days) [[22](#_ENREF_22)]. A course of chemotherapy was assumed to be six cycles. As each cycle was administered over two days we assumed a resource use of two per cycle for chemotherapy administration. We assumed a body surface area of 1.85 m² for drug resource use. Half of individuals with Stage II–III endometrial cancer were assumed to receive chemotherapy as were all individuals with Stage IV endometrial cancer [[21](#_ENREF_21)].

Unit costs for diagnostic tests were estimated by taking the mean average price of each test offered to the NHS by clinical genetics laboratories identified through the UK Genetic Testing Network [[23](#_ENREF_23)], with the exception of IHC, for which the price was provided directly by Dr Mark Arends (Department of Pathology, University of Cambridge) and Dr Ian Frayling (All-Wales Genetics Service). Unit costs for diagnostic tests nominally included overheads for administration, equipment wear and tear, training and repeat tests.

Unit costs for colonoscopies, adverse events, colorectal surgery and gynaecological surgery were estimated using the Department of Health reference costs 2011/12 [[24](#_ENREF_24)]. Unit costs for CRC treatment (except surgery) were taken from Trueman et al. [[20](#_ENREF_20)]. Unit costs for chemotherapy drugs for endometrial cancer were taken from the eMit database which gives the average price paid by the NHS for generic drugs [[25](#_ENREF_25)]. Unit costs for radiotherapy were derived from Havrilesky et al. [[21](#_ENREF_21)].

## Currency, price date and conversion

Costs are reported in 2013/14 GBP. Costs were inflated to 2011/12 prices using the Hospital and Community Health Services (HCHS) Pay and Prices Index [[26](#_ENREF_26)] and then further inflating to 2013/14 at a rate of 3.46% per annum, which was the geometric average of inflation of the index in the period 2000/01 to 2011/12.

Radiotherapy costs needed to be converted from 2009 USD and this was done using purchasing power parity and inflating to 2011 using the EPPI-Centre Cost Converter [[27](#_ENREF_27), [28](#_ENREF_28)] and then further inflating to 2013/14 prices.

## Choice of model

We used an individual patient sampling model due to the difficulty of modelling concurrent disease and service processes, as well as time-in-state-dependent transition probabilities within the more traditional Markov cohort modelling framework.

Simple interactions were included in the sense that relatives would only be eligible for genetic testing if probands were tested, but other possible interactions were not included, e.g., individuals might be more willing to take up surveillance colonoscopies if a relative developed CRC; such interactions were not modelled.

The model was event driven with continuous time. Each year the risks of applicable events would be computed and then hypothetical event times sampled. Events were divided into competing events (events resulting in death) and non-competing events (events not resulting in death). If none of the sampled times was within one year then no event would be simulated and time would advance a year. If one or more of the sampled times was within one year then the earliest competing event was simulated as well as all non-competing events occurring before the first competing event. The hazard rate of all events was assumed to be piece-wise constant each year. Events drove the patient state, which in turn defined which events were applicable and at what risk level.

Figure S1: Overview of diagnostic pathways

Figure S2: Disease states in model

Notes: CRC is further divided into rectal and colon cancer

Figure S3: Surgery for colorectal cancer

## Model assumptions

The following assumptions were explicitly made in the construction of the model. Other assumptions may be implicit; this list may not be exhaustive.

1. Diagnosis of Lynch syndrome:
   1. Diagnosis is assumed to occur instantly, i.e., no delay in diagnosing relatives, no surveillance offered before diagnosis.
   2. MSI and IHC are assumed to be diagnostically independent in Strategy 7, i.e., sensitivity and specificity of MSI testing equal for IHC-negative and IHC-naïve individuals.
   3. Diagnostic genetic testing is assumed to be diagnostically independent of previous tests.
   4. Predictive genetic testing is assumed to be 100% accurate.
   5. Variants of unknown significance are not explicitly modelled but the sensitivity of diagnostic genetic testing is conservatively set to 90% to account for this and other factors, following the example of Dinh et al. [[6](#_ENREF_6)]
2. Modelling of colorectal cancer:
   1. Individuals would be at risk of at most two colorectal cancers over their lifetime.
   2. Synchronous CRC was treated as equivalent to single CRC with Dukes’ stage equal to that of the more advanced carcinoma.
   3. The risk of mortality from separate cancers (i.e., metachronous CRC, endometrial cancer) is additive in the hazard rate.
   4. Removal of a section of the colorectum reduces the hazard of CRC incidence proportionally to the amount of CRC incidence occurring in that section in patients with Lynch syndrome; e.g., 94% of CRCs in individuals with Lynch syndrome occur in the colon,[[6](#_ENREF_6)] therefore by removing the colon (subtotal colectomy) the hazard of CRC incidence is reduced by 94%. With additional complexity it would be possible to model instead risk reduction according to Lynch syndrome status but given the very low incidence of metachronous CRC in individuals without Lynch syndrome this was assumed to have a negligible effect on cost-effectiveness.
   5. Dukes’ stage at diagnosis is dependent only on whether the individual is receiving biennial surveillance colonoscopies and is explicitly independent of: age, sex, Lynch syndrome disease status, Lynch syndrome diagnosis, whether the individual is a proband or relative, CRC history, endometrial cancer status, and previous colorectal surgery.
   6. Any simulated individuals with pre-existing CRC face no ongoing mortality from their previous CRC.
   7. Disutility from CRC is dependent only on Dukes’ stage. Notably it does not change with time from diagnosis or time until death (e.g., to represent terminal cancer).
   8. The Dukes’ stage for a CRC is fixed from diagnosis until death. The survival curves for Dukes’ stages are based on stage at diagnosis, as are the utilities. Costs for chemotherapy and surgery for cancer recurrence are modelled.
   9. Mortality from CRC is dependent on time since diagnosis, Lynch syndrome status, Dukes’ stage and patient age at diagnosis. It is explicitly independent of CRC site.
3. Modelling of endometrial cancer:
   1. Endometrial cancer is not modelled for individuals without Lynch syndrome, since incidence in the general population is low and such individuals are unlikely to be diagnosed with Lynch syndrome and offered H-BSO.
   2. A maximum of one endometrial cancer is modelled. It is assumed that treatment for endometrial cancer is total hysterectomy which has been shown to be effective at eliminating the risk of metachronous endometrial cancer [[29](#_ENREF_29)], plus bilateral salpingo-oophorectomy if Lynch syndrome has been diagnosed.
   3. Separate endometrial cancer stages are not explicitly modelled. Instead average resource use, survival and utility are applied, weighted by the proportion of patients diagnosed with each stage.
   4. Endometrial cancer stage on diagnosis is independent of all patient characteristics, including Lynch syndrome status.
   5. Disutility from endometrial cancer is constant (zero in base case). Notably it does not change with time from diagnosis or time until death.
4. Modelling of surveillance colonoscopies:
   1. Individuals within the surveillance programme do not miss or delay colonoscopies. Some colonoscopies are repeated due to failure.
   2. Individuals cannot crossover into or out of the surveillance programme.
   3. The effectiveness of surveillance colonoscopies is modelled by applying a hazard ratio for the duration of surveillance, i.e., between age 25 or the age at which the individual begins the simulation (whichever is later) and 75.
   4. Risks of adverse events are independent of patient characteristics (e.g., age, previous CRC, previous perforation).
5. Utility:
   1. Disutility from cancer is additive, i.e., utility of individual with cancer = utility of individual without cancer – disutility from cancer.
   2. Baseline utility is piece-wise constant (recomputed each year).
   3. The disutility from metachronous CRC is equal to the disutility from the more advanced CRC.
   4. No disutility from transient events, e.g., surveillance colonoscopies.
   5. Disutility due to psychological impact of genetic testing applies for 4 months.
6. Costs:
   1. Unit costs are independent of patient characteristics, e.g., segmental resection costs same for an individual with Lynch syndrome as for an individual without Lynch syndrome.
   2. The cost of surveillance colonoscopies has been reduced by a third in the base case to account for the effectiveness estimates being from studies with 3-yearly colonoscopy rather than biennial. Sensitivity analyses showed that while cost-effectiveness is sensitive to the cost of colonoscopy, even when the cost is doubled from the base case (one third above the true cost) all strategies remain cost-effective versus no testing (ICERs all below £20,000 per QALY).

## Analytical methods

### Extrapolation and interpolation of incidence and survival data

Colorectal cancer incidence in the general population was estimated by simple pooling of CRC registration statistics from 2006 to 2010 inclusive. We assumed the same incidence rate within each age group in the registration statistics (usually five-year age groups). There was no need to extrapolate as registration statistics covered ages up to 100 (the maximum age in the model).

Colorectal cancer incidence in the Lynch syndrome population was estimated by fitting a sex-specific logistic model for cumulative risk to data from Bonadona et al. [[30](#_ENREF_30)]. For each year of age constant incidence rate was assumed to match the cumulative risk function. Incidence beyond 80 years was extrapolated using the logistic model.

Endometrial cancer incidence in the Lynch syndrome population was estimated by assuming a constant incidence rate in the age groups 0–29, 30–39, 40–49, 50–59, 60–69, and 70–79 to match the cumulative risk data from Bonadona et al. [[30](#_ENREF_30)]. Zero incidence was assumed from age 80.

Colorectal cancer survival was estimated from Dukes’ stage-specific survival measured at each year for 5 years [[31](#_ENREF_31)]. A constant Dukes’ stage-specific mortality rate in each year was assumed, and the mortality rate for 4–5 years was extrapolated beyond five years. Mortality was reduced for individuals with Lynch syndrome with Dukes’ A and B CRC using a hazard ratio of 0.57 [[32](#_ENREF_32)] but was not reduced for Dukes’ C and D [[33](#_ENREF_33)]. Hazard ratios were also applied to the CRC mortality rate according to age at diagnosis, based on the age groups 0–69, 70–79 and 80+.

Endometrial cancer survival was estimated from survival measured at 1, 3, 5 and 10 years, and a piece-wise constant mortality rate was assumed to match this. Zero mortality was assumed after 10 years.

### Handling heterogeneity and uncertainty

Heterogeneity was explicitly incorporated in the individual patient sampling. In the base case, 384,000 probands and 384,000 relatives were simulated, divided equally according to whether they truly had Lynch syndrome (true OR false), their diagnosis and management (LS not diagnosed OR LS diagnosed but surveillance rejected OR LS diagnosed and surveillance accepted), and sex (male OR female). In each of these 24 groups other patient characteristics were randomly sampled. Age was sampled for all according to proband/relative status, Lynch syndrome status and sex. The initial CRC state was also sampled randomly (healthy for relatives and a Dukes’ stage for probands), as was the extent of any previous colorectal surgery. Average costs and outcomes were obtained for each of the 24 groups, effectively “integrating out” patient heterogeneity.

Uncertainty was handled through univariate sensitivity analyses and scenario analyses because the computation time for a probabilistic sensitivity analysis would have been intractable.

## Characterising uncertainty

### Univariate sensitivity analyses

We evaluated the sensitivity of results to all parameters through univariate sensitivity analysis, except in some cases where costs were grouped together, e.g., the cost of all CRC surgery types were varied simultaneously.

Figure S4 demonstrates the impact on cost-effectiveness when costs are individually halved or doubled. In reality many of the parameters are not subject to this level of uncertainty – for example the cost of prophylactic hysterectomy and bilateral salpingo-oophorectomy is well estimated – while for other parameters a large part of the range explored may be reasonable, e.g., the cost of diagnostic tests may fluctuate significantly due to technological advances. The cost of colonoscopy is clearly an important parameter for cost-effectiveness. Its value is unlikely to be subject to uncertainty, however, since many thousands of colonoscopies are performed each year in the NHS and hence its cost should be very close to the reference cost. Strategy 5 remained cost-effective versus no testing across all explored parameter values.

Figure S5 demonstrates the impact on cost-effectiveness when compliance/acceptance parameters are varied. For most of these parameters we firstly halved compliance, and secondly halved non-compliance. For all but one parameter, increased acceptance/compliance improved the cost-effectiveness of Strategy 5 versus no testing; the exception was acceptance of prophylactic H-BSO, for which increased acceptance led to worsened cost-effectiveness. Strategy 5 remained very cost-effective versus no testing across all explored parameter values.

Figure S6 demonstrates the impact of varying diagnostic test accuracy parameters on cost-effectiveness. The alternative parameter values explored cover the range seen in studies of diagnostic test accuracy, particularly those identified by Palomaki et al. [[4](#_ENREF_4)]. Strategy 5 remained very cost-effective versus no testing across all explored parameter values.

Figure S7 demonstrates the sensitivity of cost-effectiveness results to the effectiveness of surveillance colonoscopy at reducing the incidence of CRC. The range explored for the hazard ratio for index CRC incidence reflects one standard error around the mean as determined from a Cox proportional hazards analysis of data presented by Jarvinen et al.[[34](#_ENREF_34)] The range explored for the hazard ratio for metachronous CRC incidence reflects a worst-case scenario where no benefit is derived. Strategy 5 remains very cost-effective versus no testing across all explored parameter values.

Figure S8 demonstrates the impact of varying epidemiological parameters related to Lynch syndrome on cost-effectiveness. The prevalence of Lynch syndrome in the early-onset CRC population was halved and doubled and this had a significant effect on cost-effectiveness, although Strategy 5 remained cost-effective versus no testing even when the prevalence was halved. We explored the impact of the number of relatives identified per proband on cost-effectiveness by exploring the worst-case scenario (zero relatives) and an optimistic scenario (twelve relatives). Although cost-effectiveness worsened when no relatives were identified, Strategy 5 remained cost-effective.

Figure S9 demonstrates the sensitivity of cost-effectiveness results to natural history parameters of colorectal and endometrial cancer for individuals with Lynch syndrome. The uncertainty in the incidence of colorectal cancer for individuals with Lynch syndrome was investigated by varying the principle parameter for determining lifetime risk of colorectal cancer in the model, $\beta_{0}$. This was varied such that the 95% confidence interval for the cumulative risk to age 70 according to Bonadona et al.[[30](#_ENREF_30)] was explored. This had an impact on cost-effectiveness, although the resulting ICER for Strategy 5 versus no testing only marginally exceeded £10,000 per QALY in the worst case explored. Endometrial cancer incidence and colorectal cancer survival for individuals with Lynch syndrome did not have a significant impact on cost-effectiveness across the values explored.

Figure S10 demonstrates the impact of changes to utility parameters on cost-effectiveness, importantly including the impact of including a disutility for prophylactic H-BSO. Inclusion of a disutility of 0.1 results in all strategies being dominated by no testing. In the other sensitivity analyses Strategy 5 remained cost-effective versus no testing.

Figure S4: Tornado diagram showing sensitivity of results to cost parameters

**Notes:** ICERs shown are for Strategy 5 (MSI followed by BRAF followed by diagnostic genetic testing) versus no testing; the cost-effectiveness of strategies including IHC are similarly sensitive to the cost of IHC. Cost parameters are halved and doubled in their respective sensitivity analysis, with blue bars indicating that increased cost increases the ICER of Strategy 5 versus no testing, while red bars indicate a decreasing ICER.

Figure S5: Tornado diagram showing sensitivity of results to compliance/acceptance parameters

Figure S6: Tornado diagram showing sensitivity of results to diagnostic test accuracy parameters

Figure S7: Tornado diagram showing sensitivity of results to effectiveness of surveillance colonoscopy

Figure S8: Tornado diagram showing sensitivity of results to parameters relating to Lynch syndrome epidemiology

Figure S9: Tornado diagram showing sensitivity of results to natural history parameters

Figure S10: Tornado diagram showing sensitivity of results to utility parameters

## References

1. Husereau D, Drummond M, Petrou S, et al. Consolidated Health Economic Evaluation Reporting Standards (CHEERS) statement. Bmj 2013;**346**:f1049 doi: 10.1136/bmj.f1049.

2. Husereau D, Drummond M, Petrou S, et al. Consolidated Health Economic Evaluation Reporting Standards (CHEERS)--explanation and elaboration: a report of the ISPOR Health Economic Evaluation Publication Guidelines Good Reporting Practices Task Force. Value Health 2013;**16**(2):231-50 doi: 10.1016/j.jval.2013.02.002.

3. National Institute for Health and Care Excellence. Guide to the methods of technology appraisal. Process and methods guides, 2013.

4. Palomaki GE, McClain MR, Melillo S, Hampel HL, Thibodeau SN. EGAPP supplementary evidence review: DNA testing strategies aimed at reducing morbidity and mortality from Lynch syndrome. Genetics in medicine : official journal of the American College of Medical Genetics 2009;**11**(1):42-65 doi: 10.1097/GIM.0b013e31818fa2db[published Online First: 7 January 2009].

5. Bonis PA, Trikalinos TA, Chung M, et al. Hereditary nonpolyposis colorectal cancer: diagnostic strategies and their implications. Evidence report/technology assessment 2007(150):1-180 [published Online First: 2007/09/04].

6. Dinh TA, Rosner BI, Atwood JC, et al. Health Benefits and Cost-Effectiveness of Primary Genetic Screening for Lynch Syndrome in the General Population. Cancer Prevention Research 2011;**4**(1):9-22 doi: 10.1158/1940-6207.capr-10-0262[published Online First: 18 November 2010].

7. Ladabaum U, Wang G, Terdiman J, et al. Strategies to identify the Lynch syndrome among patients with colorectal cancer. Annals of Internal Medicine 2011;**155**(2):69-79 doi: 10.7326/0003-4819-155-2-201107190-00002.

8. Mvundura M, Grosse SD, Hampel H, Palomaki GE. The cost-effectiveness of genetic testing strategies for Lynch syndrome among newly diagnosed patients with colorectal cancer. Genetics in medicine : official journal of the American College of Medical Genetics 2010;**12**(2):93-104 doi: 10.1097/GIM.0b013e3181cd666c[published Online First: 15 January 2009].

9. Ara R, Brazier JE. Populating an Economic Model with Health State Utility Values: Moving toward Better Practice. Value Health 2010;**13**(5):509-18 doi: 10.1111/j.1524-4733.2010.00700.x.

10. Dolan P, Gudex C, Kind P, Williams A. The time trade-off method: results from a general population study. Health economics 1996;**5**(2):141-54 doi: 10.1002/(SICI)1099-1050(199603)5:2<141::AID-HEC189>3.0.CO;2-N.

11. Kuppermann M, Wang G, Wong S, et al. Preferences for outcomes associated with decisions to undergo or forgo genetic testing for Lynch syndrome. Cancer 2013;**119**(1):215-25 doi: 10.1002/Cncr.27634[published Online First: 11 July 2012].

12. Cooper K, Squires H, Carroll C, et al. Chemoprevention of colorectal cancer: Systematic review and economic evaluation. Health technology assessment 2010;**14**(32):1-205.

13. Ness RM, Holmes AM, Klein R, Dittus R. Utility valuations for outcome states of colorectal cancer. Am. J. Gastroenterol. 1999;**94**(6):1650-57 doi: 10.1016/s0002-9270(99)00213-0.

14. Ramsey SD, Andersen MR, Etzioni R, et al. Quality of life in survivors of colorectal carcinoma. Cancer 2000;**88**(6):1294-303 doi: 10.1002/(sici)1097-0142(20000315)88:6<1294::aid-cncr4>3.0.co;2-m[published Online First: 20 November 2000].

15. Boyle MH, Furlong W, Feeny D, Torrance GW, Hatcher J. Reliability of the Health Utilities Index--Mark III used in the 1991 cycle 6 Canadian General Social Survey Health Questionnaire. Quality of life research : an international journal of quality of life aspects of treatment, care and rehabilitation 1995;**4**(3):249-57.

16. Torrance GW, Feeny DH, Furlong WJ, Barr RD, Zhang Y, Wang Q. Multiattribute utility function for a comprehensive health status classification system. Health Utilities Index Mark 2. Medical care 1996;**34**(7):702-22.

17. Richardson J, Khan MA, Iezzi A, Maxwell A. Cross-national comparison of twelve quality of life instruments; MIC Paper 3: United Kingdom. Monash, Australia: Centre for Health Economics, Monash University, 2012.

18. Mittmann N, Au HJ, Tu DS, et al. Prospective Cost-Effectiveness Analysis of Cetuximab in Metastatic Colorectal Cancer: Evaluation of National Cancer Institute of Canada Clinical Trials Group CO.17 Trial. Journal of the National Cancer Institute 2009;**101**(17):1182-92 doi: 10.1093/jnci/djp232[published Online First: 7 August 2009].

19. Gavin DR, Valori RM, Anderson JT, Donnelly MT, Williams JG, Swarbrick ET. The national colonoscopy audit: a nationwide assessment of the quality and safety of colonoscopy in the UK. Gut 2013;**62**(2):242-9 doi: 10.1136/gutjnl-2011-301848[published Online First: 1 June 2012].

20. Trueman P, Lowson K, Bending M, et al. Bowel Cancer Services: Costs and Benefits: York Health Economics Consortium, 2007.

21. Havrilesky L, Maxwell G, Myers E. Cost-effectiveness analysis of annual screening strategies for endometrial cancer. American Journal of Obstetrics & Gynecology 2009;**200**(6):640.e1 - 40.e7.

22. Fleming GF, Brurietto VL, Cella D, et al. Phase III trial of doxorubicin plus cisplatin with or without paclitaxel plus filgrastim in advanced endometrial carcinoma: A gynecologic oncology group study. Journal of Clinical Oncology 2004;**22**(11):2159-66 doi: 10.1200/jco.2004.07.184.

23. UK Genetic Testing Network (UKGTN). UKGTN database, 2012.

24. Department of Health. NHS Reference Costs 2011-2012 (available from: <http://www.dh.gov.uk/health/2012/11/2011-12-reference-costs/)>. Secondary NHS Reference Costs 2011-2012 (available from: <http://www.dh.gov.uk/health/2012/11/2011-12-reference-costs/>) 2012.

25. CMU. Electronic Market Information Tool (eMit). Commercial Medicines Unit. <http://cmu.dh.gov.uk/electronic-market-information-tool-emit/>. Secondary Electronic Market Information Tool (eMit). Commercial Medicines Unit. <http://cmu.dh.gov.uk/electronic-market-information-tool-emit/> 2013.

26. Curtis L. Unit costs of health and social care 2012. 20th ed: Personal Social Services Research Unit, 2012.

27. EPPI. CCEMG - EPPI-Centre Cost Converter (v.1.2). <http://eppi.ioe.ac.uk/costconversion/default.aspx> 2013.

28. Shemilt I, Thomas J, Morciano M. A web-based tool for adjusting costs to a specific target currency and price year. Evidence and Policy 2010;**6**(1):51-59.

29. Schmeler KM, Lynch HT, Chen LM, et al. Prophylactic surgery to reduce the risk of gynecologic cancers in the Lynch syndrome. The New England journal of medicine 2006;**354**(3):261-9 doi: 10.1056/NEJMoa052627.

30. Bonadona V, Bonaiti B, Olschwang S, et al. Cancer risks associated with germline mutations in MLH1, MSH2, and MSH6 genes in Lynch syndrome. JAMA : the journal of the American Medical Association 2011;**305**(22):2304-10 doi: 10.1001/jama.2011.743[published Online First: 7 June 2011].

31. National Cancer Intelligence Network (NCIN). Colorectal cancer survival by stage (available from: <http://www.ncin.org.uk/publications/data_briefings/colorectal_cancer_survival_by_stage.aspx)>, 2009.

32. Lin KM, Shashidharan M, Ternent CA, et al. Colorectal and extracolonic cancer variations in MLH1/MSH2 hereditary nonpolyposis colorectal cancer kindreds and the general population. Diseases of the colon and rectum 1998;**41**(4):428-33 [published Online First: 1998/04/29].

33. Barnetson RA, Tenesa A, Farrington SM, et al. Identification and survival of carriers of mutations in DNA mismatch-repair genes in colon cancer. The New England journal of medicine 2006;**354**(26):2751-63 doi: 10.1056/NEJMoa053493[published Online First: 2006/06/30].

34. Jarvinen HJ, Aarnio M, Mustonen H, et al. Controlled 15-year trial on screening for colorectal cancer in families with hereditary nonpolyposis colorectal cancer. Gastroenterology 2000;**118**(5):829-34 [published Online First: 28 April 2000].
