# Supplementary material for: A model-based assessment of the cost–utility of strategies to identify Lynch syndrome in early-onset colorectal cancer patients
Source: BMC Cancer. 2015 Apr 25;15:313. doi: 10.1186/s12885-015-1254-5 (PMC4428233; doi:10.1186/s12885-015-1254-5)
Supplement: Additional file 3: — Supplementary Tables. Provides supplementary tables of model parameter values and sources for the decision analytic model. [file 12885_2015_1254_MOESM3_ESM.docx]

# Additional file 3 – Supplementary tables

## Table S1. Summary of model parameters (diagnostic component)

| **Model parameter** | **Base case value** | **Source** |
| --- | --- | --- |
| *Population* | | |
| Number of probands each year | 1,699 | Office for National Statistics (2012)[^1^](#_ENREF_1) |
| Prevalence of LS in probands | 8.4% | Hampel et al. (2008)[^2^](#_ENREF_2) |
| Distribution of mutations in probands with LS | 32% *MLH1*  39% *MSH2*  14% *MSH6*  15% *PMS2* | Palomaki et al. (2009)[^3^](#_ENREF_3) |
| Number of relatives | 5 per proband | Barrow et al. (2009)[^4^](#_ENREF_4) and unpublished data provided by Ian Frayling |
| Proportion of relatives who are first-degree relatives of proband | 42% | Jenkins et al. (2006)[^5^](#_ENREF_5) and Hampel et al. (2008)[^2^](#_ENREF_2) |
| Prevalence of LS in relatives tested | 44% | Jenkins et al. (2006),[^5^](#_ENREF_5) Hampel et al. (2008)[^2^](#_ENREF_2) and unpublished data provided by Ian Frayling and Munaza Ahmed |
| *Test accuracy* | | |
| Sensitivity | | |
| …Amsterdam II criteria | 39% | Pooling of five studies[^6-10^](#_ENREF_6) |
| …MSI | 89% *MLH1*  89% *MSH2*  77% *MSH6*  77% *PMS2* | Palomaki et al. (2009)[^3^](#_ENREF_3) |
| …IHC | 77.0% | Palomaki et al. (2009)[^3^](#_ENREF_3) |
| …BRAF (given MSI positive) | 100% | Domingo et al. (2004)[^11^](#_ENREF_11) |
| …BRAF (given MLH1 abnormal on IHC) | 100% | Palomaki et al. (2009)[^3^](#_ENREF_3) |
| …Diagnostic genetic testing | 90% *MLH1*  90% *MSH2*  90% *MSH6*  62% *PMS2* | Dinh et al. (2011)[^12^](#_ENREF_12) and Palomaki et al. (2009)[^3^](#_ENREF_3) |
| …Predictive genetic testing | 100% | Assumed |
| Specificity | | |
| …Amsterdam II criteria | 98% | Pooling of five studies[^6-10^](#_ENREF_6) |
| …MSI | 90.2% | Palomaki et al. (2009)[^3^](#_ENREF_3) |
| …IHC | 88.8% | Palomaki et al. (2009)[^3^](#_ENREF_3) |
| …BRAF (given MSI positive) | 40% | Domingo et al. (2004)[^11^](#_ENREF_11) |
| …BRAF (given MLH1 abnormal on IHC) | 69% | Palomaki et al. (2009)[^3^](#_ENREF_3) |
| …Diagnostic genetic testing | 99.97% | Dinh et al. (2011)[^12^](#_ENREF_12) and Palomaki et al. (2009)[^3^](#_ENREF_3) |
| …Predictive genetic testing | 100% | Assumed |
| Distribution of IHC false positive results | 90% *MLH1*  6% *MSH2*  2% *MSH6*  2% *PMS2* | Mvundura et al. (2010)[^13^](#_ENREF_13) |
| *Acceptance of stages of diagnosis* | | |
| Acceptance of MSI testing | 100% | Ramsey et al. (2003)[^14^](#_ENREF_14) |
| Acceptance of IHC, BRAF | 100% | Assumed |
| Acceptance of genetic counselling (proband) | 92.5% | Clinical expert (Ian Frayling) opinion “90 to 95 per cent” |
| Acceptance of diagnostic genetic testing (first test) | 90% | Ladabaum et al. (2011)[^15^](#_ENREF_15) |
| Acceptance of family history assessment when genetic counselling or diagnostic genetic testing declined (proband) | 100% | Assumed |
| Acceptance of genetic counselling (relatives) | 45% | Calculated using data from Palomaki et al. (2009)[^3^](#_ENREF_3) |
| Acceptance of predictive genetic testing | 96% | Calculated using data from Palomaki et al. (2009)[^3^](#_ENREF_3) |
| *Costs (GBP, 2013/14 prices)* | | |
| MSI | £202 | Average of Oxford Medical Genetics Laboratories,[^16^](#_ENREF_16) All Wales Molecular Genetics Laboratory,[^17^](#_ENREF_17) West Midlands Regional Genetics Laboratory (via the UKGTN)[^18^](#_ENREF_18) |
| IHC | £238 | Dr Mark Arends (Department of Pathology, University of Cambridge) and Dr Ian Frayling (on behalf of All-Wales Genetics Service) |
| BRAF | £118 | Average of personal communication with Mr Michael Gandy (UCL-Advanced Diagnostics), East of Scotland Regional Genetic Service (previously available online, no longer available), All Wales Molecular Genetics Laboratory[^17^](#_ENREF_17) |
| Combined diagnostic genetic test MLH1, MSH2 and MSH6 | £812 | Average of Oxford Medical Genetics Laboratories,[^16^](#_ENREF_16) All Wales Molecular Genetics Laboratory,[^17^](#_ENREF_17) East Anglian Medical Genetics Laboratories,[^19^](#_ENREF_19) Yorkshire Regional Genetics Service (previously available online, no longer available) |
| Diagnostic genetic test PMS2 | £735 | Yorkshire Regional Genetics Service (previously available online, no longer available) |
| Diagnostic genetic test MLH1 | £464 | Average of All Wales Molecular Genetics Laboratory,[^17^](#_ENREF_17) East of Scotland Regional Genetic Service (previously available online, no longer available), East Anglian Medical Genetics Laboratories,[^19^](#_ENREF_19) Yorkshire Regional Genetics Service (previously available online, no longer available) |
| Proband genetic counselling | £67 | The PSSRU[^20^](#_ENREF_20) and personal communication with Professor Mary Porteous (SE Scotland Genetic Service) |
| Taking family history | £22 | The PSSRU[^20^](#_ENREF_20) and personal communication with Professor Mary Porteous (SE Scotland Genetic Service) |
| Predictive genetic test MLH1 | £169 | Average of Oxford Medical Genetics Laboratories,[^16^](#_ENREF_16) All Wales Molecular Genetics Laboratory,[^17^](#_ENREF_17) East of Scotland Regional Genetic Service (previously available online, no longer available), East Anglian Medical Genetics Laboratories,[^19^](#_ENREF_19) Yorkshire Regional Genetics Service (previously available online, no longer available) |
| Predictive genetic test MSH2 or MSH6 | £172 | Average of Oxford Medical Genetics Laboratories,[^16^](#_ENREF_16) All Wales Molecular Genetics Laboratory,[^17^](#_ENREF_17) Yorkshire Regional Genetics Service (previously available online, no longer available) |
| Predictive genetic test PMS2 | £176 | Yorkshire Regional Genetics Service (previously available online, no longer available) |
| Relative genetic counselling | £67 | The PSSRU[^20^](#_ENREF_20) and personal communication with Professor Mary Porteous (SE Scotland Genetic Service) |
| *Disutility due to psychological impact of genetic testing* | | |
| Proband | | |
| …Test declined | | |
| ……H-BSO not offered | 0.04 | Kuppermann et al. (2013)[^21^](#_ENREF_21) |
| ……H-BSO accepted | 0.05 | Kuppermann et al. (2013)[^21^](#_ENREF_21) |
| ……H-BSO declined | 0.11 | Kuppermann et al. (2013)[^21^](#_ENREF_21) |
| …Test accepted | | |
| ……LS −ve | 0.00 | Assumed |
| ……LS +ve | | |
| ………H-BSO not offered | 0.02 | Kuppermann et al. (2013)[^21^](#_ENREF_21) |
| ………H-BSO accepted | 0.03 | Kuppermann et al. (2013)[^21^](#_ENREF_21) |
| ………H-BSO declined | 0.09 | Kuppermann et al. (2013)[^21^](#_ENREF_21) |
| Relative | | |
| …Test declined | | |
| ……H-BSO not offered | 0.04 | Kuppermann et al. (2013)[^21^](#_ENREF_21) |
| ……H-BSO accepted | 0.08 | Kuppermann et al. (2013)[^21^](#_ENREF_21) |
| ……H-BSO declined | 0.11 | Kuppermann et al. (2013)[^21^](#_ENREF_21) |
| …Test accepted | | |
| ……LS −ve | 0.00 | Assumed |
| ……LS +ve | | |
| ………H-BSO not offered | 0.02 | Kuppermann et al. (2013)[^21^](#_ENREF_21) |
| ………H-BSO accepted | 0.06 | Kuppermann et al. (2013)[^21^](#_ENREF_21) |
| ………H-BSO declined | 0.09 | Kuppermann et al. (2013)[^21^](#_ENREF_21) |
| Duration of disutility | 4 months | Assumption based on Heshka et al. (2008)[^22^](#_ENREF_22) |

## Table S2. Summary of model parameters (management component)

| **Model parameter** | **Base case value** | **Source** |
| --- | --- | --- |
| *CRC natural history* | | |
| Cumulative risk of CRC (to age 70 years) | | |
| …Without LS | | |
| ……Male | 2.8% | Office for National Statistics[^1^](#_ENREF_1)^,^ [^23-26^](#_ENREF_23) |
| ……Female | 1.8% | Office for National Statistics[^1^](#_ENREF_1)^,^ [^23-26^](#_ENREF_23) |
| …With LS | | |
| ……Male | 38.3% | Logistic curve fitted to Bonadona et al. (2011)[^27^](#_ENREF_27) |
| ……Female | 31.2% | Logistic curve fitted to Bonadona et al. (2011)[^27^](#_ENREF_27) |
| Dukes’ stage on diagnosis | 16.4% Dukes’ A  31.7% Dukes’ B  27.1% Dukes’ C  24.8% Dukes’ D | Finan et al. (2011)[^28^](#_ENREF_28) |
| CRC site (colon/rectum) | | |
| …Without LS | | |
| ……Male | 58/42 | Office for National Statistics[^1^](#_ENREF_1) |
| ……Female | 61/39 | Office for National Statistics[^1^](#_ENREF_1) |
| …With LS | 94/6 | Dinh et al. (2011)[^12^](#_ENREF_12) |
| Relative CRC survival (Without LS) | | |
| …Dukes’ A | 1 year: 0.969  3 year: 0.957  5 year: 0.932 | National Cancer Intelligence Network[^29^](#_ENREF_29) |
| …Dukes’ B | 1 year: 0.917  3 year: 0.831  5 year: 0.770 | National Cancer Intelligence Network[^29^](#_ENREF_29) |
| …Dukes’ C | 1 year: 0.815  3 year: 0.583  5 year: 0.477 | National Cancer Intelligence Network[^29^](#_ENREF_29) |
| …Dukes’ D | 1 year: 0.380  3 year: 0.116  5 year: 0.066 | National Cancer Intelligence Network[^29^](#_ENREF_29) |
| Hazard ratio for CRC mortality due to age at diagnosis | | |
| …Under 70y | Year 1: 0.599  Years 2-5: 0.972 | Office for National Statistics[^30^](#_ENREF_30) |
| …70–79y | Year 1: 0.956  Years 2-5: 0.966 | Office for National Statistics[^30^](#_ENREF_30) |
| …80y and over | Year 1: 1.797  Years 2-5: 1.116 | Office for National Statistics[^30^](#_ENREF_30) |
| Hazard ratio for CRC mortality due to LS | | |
| …Dukes’ A/B | 0.57 | Lin et al. (1998)[^31^](#_ENREF_31) |
| …Dukes’ C/D | 1 | Barnetson et al. (2006)[^7^](#_ENREF_7) |
| *EC natural history* | | |
| Cumulative risk of EC for women with LS (to age 70 years) | 34% | Bonadona et al. (2011)[^27^](#_ENREF_27) |
| EC relative survival | 1 year: 0.901  3 year: 0.814  5 year: 0.773  10 year: 0.745 | Office for National Statistics,[^30^](#_ENREF_30) NCIN UK Cancer e-Atlas,[^32^](#_ENREF_32) Cancer Research UK[^33^](#_ENREF_33) |
| *Population characteristics* | | |
| Age distribution | | |
| …Probands | | |
| ……Without LS | To match age distribution of CRC incidence under 50 years | Office for National Statistics[^1^](#_ENREF_1)^,^ [^23-26^](#_ENREF_23) |
| ……With LS | To match age distribution of CRC incidence under 50 years | Bonadona et al. (2011)[^27^](#_ENREF_27) |
| …Relatives | | |
| ……Without LS | To match age distribution of population aged 18–75 | Office for National Statistics[^34^](#_ENREF_34)^,^ [^35^](#_ENREF_35) |
| ……With LS | As above but incorporating excess CRC mortality due to LS | Modelled |
| *Effectiveness of surveillance colonoscopies* | | |
| Hazard ratio for CRC incidence due to biennial surveillance colonoscopy | | |
| …First CRC | 0.387 | Cox proportional hazards regression of Figure 1 from Jarvinen et al. (2000)[^36^](#_ENREF_36) |
| …Metachronous CRC | 0.533 | Calculated from Cirillo et al. (2012)[^37^](#_ENREF_37) |
| Age for surveillance colonoscopies | 25–75 | Cairns et al. (2010)[^38^](#_ENREF_38) |
| Dukes’ stage on diagnosis for patients receiving biennial surveillance colonoscopy | 68.6% Dukes’ A  10.5% Dukes’ B  12.8% Dukes’ C  8.1% Dukes’ D | Mecklin et al. (2007)[^39^](#_ENREF_39) |
| *Effectiveness of risk-reducing surgery* | | |
| Hazard ratio for CRC incidence due to risk-reducing surgery | | |
| …Segmental resection | 1.00 | (Reference) |
| …Subtotal colectomy and ileorectal anastomosis | 0.06 | Derived from Dinh et al. (2011)[^12^](#_ENREF_12) |
| …Rectal excision | 0.94 | Derived from Dinh et al. (2011)[^12^](#_ENREF_12) |
| …Proctocolectomy | 0 | Derived from Dinh et al. (2011)[^12^](#_ENREF_12) |
| Hazard ratio for EC incidence due to total hysterectomy and bilateral salpingo-oophorectomy | 0 | Schmeler et al. (2006)[^40^](#_ENREF_40) |
| *Adverse events* | | |
| Relating to colonoscopies | | |
| …Probability of bleeding (each colonoscopy) | 0.0026 | Gavin et al. (2013)[^41^](#_ENREF_41) |
| ……Probability bleeding leads to admission | 0.21 | Gavin et al. (2013)[^41^](#_ENREF_41) |
| ………Severity of bleeding leading to admission | Mild 0.73  Moderate 0.18  Severe 0.09 | Gavin et al. (2013)[^41^](#_ENREF_41) |
| …Probability of perforation (each colonoscopy) | 0.0004 | Gavin et al. (2013)[^41^](#_ENREF_41) |
| …Probability of mortality (each colonoscopy) | 0.000083 | Cairns et al. (2010)[^38^](#_ENREF_38) |
| Probability of mortality due to prophylactic hysterectomy and bilateral salpingo-oophorectomy | 0.0002 | Palomaki et al. (2009)[^3^](#_ENREF_3) |
| *Acceptance of risk-reducing measures* | | |
| Acceptance of prophylactic hysterectomy and bilateral salpingo-oophorectomy (offered to women with LS at or after age 45) | 0.55 | Personal communication, Lorraine Cowley, Northern Genetic Service |
| Acceptance of biennial surveillance colonoscopy | | |
| …Proband | | |
| ……LS mutation found | 0.8 | Ladabaum et al. (2011)[^15^](#_ENREF_15) |
| ……LS assumed | 0.7 | Ladabaum et al. (2011)[^15^](#_ENREF_15) |
| …Relative | | |
| ……LS mutation found | 0.8 | Ladabaum et al. (2011)[^15^](#_ENREF_15) |
| ……LS assumed | 0.5 | Ladabaum et al. (2011)[^15^](#_ENREF_15) |
| Proportion of individuals with LS receiving more aggressive surgery for CRC | 0 | Assumed in absence of clinical consensus |
| *Utility* | | |
| Baseline utility (*m* = 1 for male, 0 for female; *a* = age in years) | 0.9508566  + 0.0212126 *m*  − 0.002587 *a*  − 0.0000332 *a*^2^ | Ara and Brazier (2010)[^42^](#_ENREF_42) |
| Disutility due to CRC | 0.00 Dukes’ A  0.00 Dukes’ B  0.00 Dukes’ C  0.13 Dukes’ D | Ramsey et al. (2000)[^43^](#_ENREF_43) and Mittmann et al. (2009)[^44^](#_ENREF_44) |
| Disutility due to choice of CRC surgery | 0 | Assumed |
| Disutility due to EC | 0 | Assumed |
| Disutility due to prophylactic hysterectomy and bilateral salpingo-oophorectomy | 0 | Assumed |
| Disutility due to biennial surveillance colonoscopies | 0 | Assumed |
| *Costs (£GBP, 2013/14 prices)* | | |
| Colonoscopy | £395 | Department of Health Reference Costs 2011/12[^45^](#_ENREF_45) reduced by a third (see Section 6.4) |
| Adverse events relating to colonoscopy | | |
| …Mild bleeding not requiring admission | £0 | Assumption |
| …Mild bleeding requiring admission | £318 | Whyte et al. (2012)[^46^](#_ENREF_46) |
| …Moderate bleeding | £490 | Department of Health Reference Costs 2011/12[^45^](#_ENREF_45) |
| …Severe bleeding | £1,984 | Department of Health Reference Costs 2011/12[^45^](#_ENREF_45) |
| …Perforation | £5,134 | Department of Health Reference Costs 2011/12[^45^](#_ENREF_45) |
| CRC diagnosis | £499 | Trueman et al. (2007)[^47^](#_ENREF_47) |
| Primary chemotherapy and radiotherapy (colon cancer) | £0 Dukes’ A  £5,755 Dukes’ B  £13,133 Dukes’ C  £13,133 Dukes’ D | Trueman et al. (2007)[^47^](#_ENREF_47) |
| Primary chemotherapy and radiotherapy (rectal cancer) | £0 Dukes’ A  £2,848 Dukes’ B  £7,628 Dukes’ C  £7,628 Dukes’ D | Trueman et al. (2007)[^47^](#_ENREF_47) |
| Follow-up surveillance (max. 5 years from diagnosis) | £269 (colon)  £256 (rectal) | Trueman et al. (2007)[^47^](#_ENREF_47) |
| Recurrence surgery and chemotherapy (in last year of life if patient dies of CRC within 5 years of diagnosis) | £12,578 (colon)  £12,216 (rectal) | Trueman et al. (2007)[^47^](#_ENREF_47) |
| Stoma care (annual cost) | £1,684 for 11% of colon cancer patients and 49% of rectal cancer patients | Trueman et al. (2007)[^47^](#_ENREF_47) |
| Palliative care (in last year of life if patient dies of CRC) | £10,141 (colon)  £9,236 (rectal) | Trueman et al. (2007)[^47^](#_ENREF_47) |
| CRC surgery | | |
| …Segmental resection | £6,154 (LS)  £6,104 (no LS) | Department of Health Reference Costs 2011/12[^45^](#_ENREF_45) |
| …Subtotal colectomy and ileorectal anastomosis | £7,331 | Department of Health Reference Costs 2011/12[^45^](#_ENREF_45) |
| …Anterior resection | £7,399 | Department of Health Reference Costs 2011/12[^45^](#_ENREF_45) |
| …Proctocolectomy and ileal pouch anal anastomosis | £7,441 | Department of Health Reference Costs 2011/12[^45^](#_ENREF_45) |
| Prophylactic hysterectomy and bilateral salpingo-oophorectomy | £3,104 | Department of Health Reference Costs 2011/12[^45^](#_ENREF_45) |
| Endometrial cancer surgery | £3,877 | Department of Health Reference Costs 2011/12[^45^](#_ENREF_45) |
| Endometrial cancer radiotherapy | £5,909 for 47% of patients | Havrilesky et al. (2009)[^48^](#_ENREF_48) |
| Endometrial cancer chemotherapy | £3,005 for 18% of patients | Various |

## References

1. Office for National Statistics. Cancer statistics registrations, England (Series MB1) - No. 41, 2010 <http://www.ons.gov.uk/ons/rel/vsob1/cancer-statistics-registrations--england--series-mb1-/no--41--2010/index.html>, 2012.

2. Hampel H, Frankel WL, Martin E, et al. Feasibility of screening for Lynch syndrome among patients with colorectal cancer. J Clin Oncol 2008;26:5783-8.

3. Palomaki GE, McClain MR, Melillo S, et al. EGAPP supplementary evidence review: DNA testing strategies aimed at reducing morbidity and mortality from Lynch syndrome. Genet Med 2009;11:42-65.

4. Barrow E, Robinson L, Alduaij W, et al. Cumulative lifetime incidence of extracolonic cancers in Lynch syndrome: a report of 121 families with proven mutations. Clin Genet 2009;75:141-9.

5. Jenkins MA, Baglietto L, Dowty JG, et al. Cancer risks for mismatch repair gene mutation carriers: a population-based early onset case-family study. Clin Gastroenterol Hepatol 2006;4:489-98.

6. Balmana J, Balaguer F, Castellvi-Bel S, et al. Comparison of predictive models, clinical criteria and molecular tumour screening for the identification of patients with Lynch syndrome in a population-based cohort of colorectal cancer patients. J Med Genet 2008;45:557-63.

7. Barnetson RA, Tenesa A, Farrington SM, et al. Identification and survival of carriers of mutations in DNA mismatch-repair genes in colon cancer. N Engl J Med 2006;354:2751-63.

8. Green RC, Parfrey PS, Woods MO, et al. Prediction of Lynch syndrome in consecutive patients with colorectal cancer. J Natl Cancer Inst 2009;101:331-40.

9. Hampel H, Frankel WL, Martin E, et al. Screening for the Lynch syndrome (hereditary nonpolyposis colorectal cancer). N Engl J Med 2005;352:1851-60.

10. Salovaara R, Loukola A, Kristo P, et al. Population-based molecular detection of hereditary nonpolyposis colorectal cancer. Journal of clinical oncology : official journal of the American Society of Clinical Oncology 2000;18:2193-200.

11. Domingo E, Laiho P, Ollikainen M, et al. BRAF screening as a low-cost effective strategy for simplifying HNPCC genetic testing. J Med Genet 2004;41:664-668.

12. Dinh TA, Rosner BI, Atwood JC, et al. Health Benefits and Cost-Effectiveness of Primary Genetic Screening for Lynch Syndrome in the General Population. Cancer Prevention Research 2011;4:9-22.

13. Mvundura M, Grosse SD, Hampel H, et al. The cost-effectiveness of genetic testing strategies for Lynch syndrome among newly diagnosed patients with colorectal cancer. Genet Med 2010;12:93-104.

14. Ramsey SD, Burke W, Clarke L. An economic viewpoint on alternative strategies for identifying persons with hereditary nonpolyposis colorectal cancer. Genetics in Medicine 2003;5:353-363.

15. Ladabaum U, Wang G, Terdiman J, et al. Strategies to identify the Lynch syndrome among patients with colorectal cancer. Annals of Internal Medicine 2011;155:69-79.

16. Oxford Medical Genetics Laboratories. Current Disease Services [available from [http://www.ouh.nhs.uk/services/referrals/genetics/genetics-laboratories/molecular-genetics-laboratory/documents/Disease_Services-Price-List.pdf]](http://www.ouh.nhs.uk/services/referrals/genetics/genetics-laboratories/molecular-genetics-laboratory/documents/Disease_Services-Price-List.pdf%5d). Oxford: Oxford University Hospitals, 2011.

17. John M. All Wales Molecular Genetics Laboratory Service Price List [available from [http://www.wales.nhs.uk/sites3/Documents/525/MI-MGN-TestPrice_5.1.pdf]](http://www.wales.nhs.uk/sites3/Documents/525/MI-MGN-TestPrice_5.1.pdf%5d). Cardiff: All Wales Molecular Genetics Laboratory, 2012.

18. UK Genetic Testing Network (UKGTN). UKGTN database, 2012.

19. East Anglian Medical Genetics Laboratories. Services and tests available [available from: [http://www.cuh.org.uk/addenbrookes/services/clinical/genetics/genetics_labs/services_test/services_test_index.html]](http://www.cuh.org.uk/addenbrookes/services/clinical/genetics/genetics_labs/services_test/services_test_index.html%5d). Cambridge: Cambridge University Hospitals, 2012.

20. Curtis L. Unit costs of health and social care 2012. 20th ed: Personal Social Services Research Unit, 2012.

21. Kuppermann M, Wang G, Wong S, et al. Preferences for outcomes associated with decisions to undergo or forgo genetic testing for Lynch syndrome. Cancer 2013;119:215-225.

22. Heshka JT, Palleschi C, Howley H, et al. A systematic review of perceived risks, psychological and behavioral impacts of genetic testing. Genetics in Medicine 2008;10:19-32.

23. Office for National Statistics. Cancer statistics registrations, England (Series MB1) - No. 37, 2006 <http://www.ons.gov.uk/ons/rel/vsob1/cancer-statistics-registrations--england--series-mb1-/no--37--2006/index.html>, 2008.

24. Office for National Statistics. Cancer statistics registrations, England (Series MB1) - No. 39, 2008 <http://www.ons.gov.uk/ons/rel/vsob1/cancer-statistics-registrations--england--series-mb1-/no--39--2008/index.html>, 2010.

25. Office for National Statistics. Cancer statistics registrations, England (Series MB1) - No. 38, 2007 <http://www.ons.gov.uk/ons/rel/vsob1/cancer-statistics-registrations--england--series-mb1-/no--38--2007/index.html>, 2010.

26. Office for National Statistics. Cancer statistics registrations, England (Series MB1) - No. 40, 2009 <http://www.ons.gov.uk/ons/rel/vsob1/cancer-statistics-registrations--england--series-mb1-/no--40--2009/index.html>, 2011.

27. Bonadona V, Bonaiti B, Olschwang S, et al. Cancer risks associated with germline mutations in MLH1, MSH2, and MSH6 genes in Lynch syndrome. JAMA 2011;305:2304-10.

28. Finan P, Smith J, Walker K, et al. National Bowel Cancer Audit annual report, 2011.

29. National Cancer Intelligence Network (NCIN). Colorectal cancer survival by stage (available from: <http://www.ncin.org.uk/publications/data_briefings/colorectal_cancer_survival_by_stage.aspx)>, 2009.

30. Office for National Statistics. Cancer survival in England - patients diagnosed 2005-2009 and followed up to 2010 <http://www.ons.gov.uk/ons/publications/re-reference-tables.html?edition=tcm%3A77-239726>, 2011.

31. Lin KM, Shashidharan M, Ternent CA, et al. Colorectal and extracolonic cancer variations in MLH1/MSH2 hereditary nonpolyposis colorectal cancer kindreds and the general population. Dis Colon Rectum 1998;41:428-33.

32. National Cancer Intelligence Network (NCIN), UK Cancer Information Service (UKCIS). UK Cancer e-Atlas, 2011.

33. Cancer Research UK. Uterine cancer survival statistics. Volume 2012, 2012.

34. Office for National Statistics. Mid-2010 Population Estimates: England and Wales; estimated resident population by single year of age and sex <http://www.ons.gov.uk/ons/rel/pop-estimate/population-estimates-for-uk--england-and-wales--scotland-and-northern-ireland/mid-2010-population-estimates/rft---mid-2010-population-estimates.zip>, 2011.

35. Office for National Statistics. Mid-2002 to mid-2010 population estimates of the very elderly (including centenarians) England and Wales; estimated resident population <http://www.ons.gov.uk/ons/rel/mortality-ageing/population-estimates-of-the-very-elderly/2010/ew-eve-2010.xls>, 2011.

36. Jarvinen HJ, Aarnio M, Mustonen H, et al. Controlled 15-year trial on screening for colorectal cancer in families with hereditary nonpolyposis colorectal cancer. Gastroenterology 2000;118:829-34.

37. Cirillo L, Urso ED, Parrinello G, et al. High Risk of Rectal Cancer and of Metachronous Colorectal Cancer in Probands of Families Fulfilling the Amsterdam Criteria. Ann Surg 2012.

38. Cairns SR, Scholefield JH, Steele RJ, et al. Guidelines for colorectal cancer screening and surveillance in moderate and high risk groups (update from 2002). Gut 2010;59:666-89.

39. Mecklin JP, Aarnio M, Laara E, et al. Development of colorectal tumors in colonoscopic surveillance in Lynch syndrome. Gastroenterology 2007;133:1093-8.

40. Schmeler KM, Lynch HT, Chen LM, et al. Prophylactic surgery to reduce the risk of gynecologic cancers in the Lynch syndrome. N Engl J Med 2006;354:261-9.

41. Gavin DR, Valori RM, Anderson JT, et al. The national colonoscopy audit: a nationwide assessment of the quality and safety of colonoscopy in the UK. Gut 2013;62:242-9.

42. Ara R, Brazier JE. Populating an Economic Model with Health State Utility Values: Moving toward Better Practice. Value in Health 2010;13:509-518.

43. Ramsey SD, Andersen MR, Etzioni R, et al. Quality of life in survivors of colorectal carcinoma. Cancer 2000;88:1294-1303.

44. Mittmann N, Au HJ, Tu DS, et al. Prospective Cost-Effectiveness Analysis of Cetuximab in Metastatic Colorectal Cancer: Evaluation of National Cancer Institute of Canada Clinical Trials Group CO.17 Trial. Journal of the National Cancer Institute 2009;101:1182-1192.

45. Department of Health. NHS Reference Costs 2011-2012 (available from: <http://www.dh.gov.uk/health/2012/11/2011-12-reference-costs/)>. Volume 2013, 2012.

46. Whyte S, Chilcott J, Halloran S. Reappraisal of the options for colorectal cancer screening in England. Colorectal Disease 2012;14:e547–e561.

47. Trueman P, Lowson K, Bending M, et al. Bowel Cancer Services: Costs and Benefits: York Health Economics Consortium, 2007.

48. Havrilesky L, Maxwell G, Myers E. Cost-effectiveness analysis of annual screening strategies for endometrial cancer. American Journal of Obstetrics & Gynecology 2009;200:640.e1 - 640.e7.
